# RETRACTION: A Decrease of Brain MicroRNA-122 Level Is an Early Marker of Cerebrovascular Disease in the Stroke-Prone Spontaneously Hypertensive Rat

**DOI:** 10.1155/omcl/9871873

**Published:** 2025-11-18

**Authors:** Oxidative Medicine and Cellular Longevity

RETRACTION: R. Stanzione, F. Bianchi, M. Cotugno, S. Marchitti, M. Forte, C. Busceti, L. Ryskalin, F. Fornai, M. Volpe, S. Rubattu, “A Decrease of Brain MicroRNA-122 Level Is an Early Marker of Cerebrovascular Disease in the Stroke-Prone Spontaneously Hypertensive Rat,” *Oxidative Medicine and Cellular Longevity*, 2017, 1206420, https://doi.org/10.1155/2017/1206420.

The above article, published online on 18 June 2017 in Wiley Online Library (wileyonlinelibrary.com), has been retracted by agreement between the journal Editor-in-Chief, Jeannette Vasquez-Vivar, and John Wiley & Sons Ltd. The retraction has been agreed due to figure concerns, also raised on PubPeer [1]. On further investigation, additional concerns were identified with Figures 1 and 2:

• In Figure 1c, the SHRSP/JD 4 wks NF-kB p65 bands and the SHRSP/RD 4 wks ß-actin bands appear similar when flipped horizontally and resized.
• In Figure 1c, the ß-actin bands appear similar to the eNOS bands shown in the right hand side blots of Figure 2d.
• In Figure 1c, the SHRSP/JD 4 wks NF-kB p65 bands and the SHRSP/RD 4 wks eNOS bands shown in Figure 2d appear similar when flipped horizontally and resized.
• In Figure 2c, the two sets of ß-actin bands appear identical.
• In the SHRSR/JD 4 wks blots in Figure 2a, there are unexpected repeated elements in the background of the vWF bands.
• In Figure 2b, the two sets of ß-actin bands appear similar when vertically flipped and resized.
• In Figure 2d:
  ⚬ The second and third SHRSR/RD 4 wks bands shown for p-eNOS appear identical.
  ⚬ The third and fourth SHRSR/JD 4 wks bands shown for eNOS appear identical.
  ⚬ The second and third SHRSP/RD 4 wks bands shown for p-eNOS appear identical.
  ⚬ There are unexpected repeated elements in the background of the left-hand side p-eNOS blots.

The authors responded to the concerns, but could not provide the original western blots. After assessment by the Chief Editor, the results are deemed no longer reliable and the article is retracted.

The authors did not respond when asked to agree to the retraction.
